# Are the Clinical Presentations (Phenotypes) of Gitelman’s and Bartter’s Syndromes Gene Mutations Driven by Their Effects on Intracellular pH, Their “pH” Enotype?

**DOI:** 10.3390/ijms21165660

**Published:** 2020-08-07

**Authors:** Lorenzo A Calò, Paul A Davis

**Affiliations:** 1 Nephrology, Dialysis and Transplantation Unit, Department of Medicine, University of Padova, 35128 Padova, Italy; 2 Department of Nutrition, University of California at Davis, Davis, CA 95616, USA; padavis@ucdavis.edu

**Keywords:** Gitelman’s syndrome, Bartter’s syndrome, gene mutations, glycosylation, endosome pH, phenotype, ACE2, Ang 1-7

## Abstract

Gitelman’s syndrome (GS) and Bartter’s syndrome (BS) are rare inherited salt-losing tubulopathies whose variations in genotype do not correlate well with either clinical course or electrolyte requirements. Using GS/BS patients as nature’s experiments, we found them to be a human model of endogenous Ang II antagonism with activated Renin-Angiotensin System (RAS), resulting in high Ang II levels with blunted cardiovascular effects. These patients are also characterized by increased and directly correlated levels of both Angiotensin Converting Enzyme 2 (ACE2) and Ang 1-7. Understanding the myriad of distinctive and frequently overlapping clinical presentations of GS/BS arises remains challenging. Efforts to find a treatment for COVID-19 has fueled a recent surge in interest in chloroquine/hydroxychloroquine and its effects. Of specific interest are chloroquine/hydroxychloroquine’s ability to inhibit SARS-CoV infection by impairing ACE2, the SARS-CoV2 entry point, through terminal glycosylation via effects on TGN/post-Golgi pH homeostasis. Several different studies with a GS or a BS phenotype, along with a nonsyndromic form of X-linked intellectual disability linked to a mutated SLC9A7, provide additional evidence that specific gene defects can act via misregulation of TGN/post-Golgi pH homeostasis, which leads to a common mechanistic basis resulting in overlapping phenotypes. We suggest that linkage between the specific gene defects identified in GS and BS and the myriad of distinctive and frequently overlapping clinical findings may be the result of aberrant glycosylation of ACE2 driven by altered TGN/endosome system acidification caused by the metabolic alkalosis brought about by these salt-losing tubulopathies in addition to their altered intracellular calcium signaling due to a blunted second messenger induced intracellular calcium release that is, in turn, amplified by the RAS system.

## 1. Introduction

### 1.1. Gitelman’s Syndrome

Gitelman’s syndrome (GS) is a genetic tubulopathy caused by loss-of-function mutations in the SLC12A3 gene, which encodes the Na^+^-Cl^−^ cotransporter and is characterized by hypokalemic metabolic alkalosis, hypocalciuria, hypomagnesemia, activated Renin-Angiotensin System (RAS) and high Angiotensin II (Ang II) levels. However, Ang II cardiovascular effects are blunted as GS patients are either normo- or hypotensive and represent a model of endogenous Ang II antagonism [1,2].

**Figure 1:**
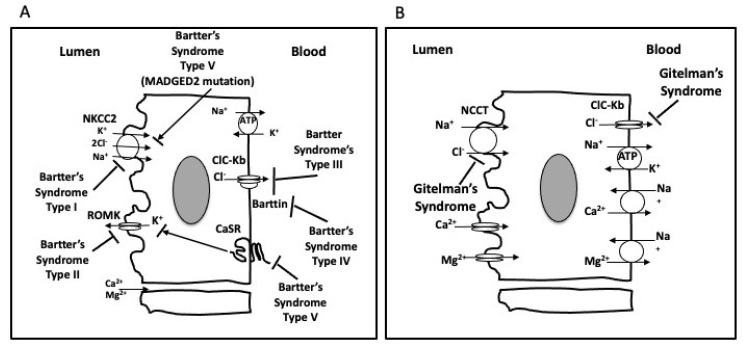
Transport pathways in the thick ascending limb of Henle’s loop depicting the five (I–V) types of Bartter’s syndrome (BS, **A**). Transport pathways in the distal convoluted tubule depicting the abnormality of Gitelman’s syndrome (GS, **B**).

GS resembles the abnormalities induced by thiazide diuretics (inhibitors of NaCl cotransporter in the distal convoluted tubule of the nephron), thereby suggesting NCC/SLC12A3 gene as a possible candidate. Mutations have been found to result in the loss of NCC/SLC12A3 function inducing NaCl wasting, hypovolemia, and metabolic alkalosis [3,4] (Figure 1), making it an autosomal recessive disease as well. Despite the growing number of causative mutations identified, up to 40% of patients are still found to carry only one SLC12A3 mutant allele; therefore, large genomic rearrangement may account for unidentified mutations [5].

### 1.2. Bartter’s Syndrome

Bartter’s syndrome (BS) includes five different types of inherited salt-losing tubulopathies all characterized by hypokalemia, hypochloremic metabolic alkalosis, activated RAS, high Ang II levels yet normotension or hypotension, and a blunted cardiovascular effect of Ang II [1] (Figure 1).

The electrolyte abnormalities of BS are similar to those induced by treatment with furosemide or other drugs that inhibit the Na-K-2Cl cotransporter of the thick ascending limb of Henle’s loop, which prompted investigations of the gene NKCC2/SLC12A1 encoding the Na-K-2Cl cotransporter. Variants of the coding region were identified in affected patients, which resulted in loss of the cotransporter function, along with Na and K wasting in the thick ascending limb of Henle’s loop and hypovolemia. These patients were thereafter classified as BS type 1. In patients in whom NKCC2 mutations were not detected, loss-of-function mutations in the apical ATP-sensitive K channel (renal outer medullary potassium channel, ROMK) were found and classified as BS type 2. As ROMK recycles K from the cell back into the lumen, the resultant fall of luminal K shuts down Na-K-2Cl cotransporter activity, resulting in salt wasting and hypovolemia, the same phenotype as BS type 1. Some patients with a BS phenotype show mutations in neither NKCC2 nor ROMK genes, but rather in the chloride channel CLCNKb. The chloride channel CLCNKb mediates Cl reabsorption across the basolateral membrane of the renal tubular cells, and the resulting intracellular chloride accumulation inhibits Na-K-2Cl cotransporter activity and manifests in salt-wasting and hypovolemia. These patients are classified as BS type 3. Two additional genetic changes have been identified as conferring a BS phenotype. BS type 4a results from a mutation of the regulatory protein Barttin (BSND) required for location of the basolateral membrane Cl channels, ClCKb and ClCKa, and BS type 4b results from a deletion mutation affecting both ClCKb and ClCKa, which are adjacent on chromosome 1. The final consequence is, as noted above for Bartter’s syndrome type III, intracellular chloride accumulation, which inhibits Na-K-2Cl cotransporter activity with ensuing salt wasting and hypovolemia. Finally, mutations in MAGED2 have been identified in a severe antenatal form of BS (BS type 5), which spontaneously resolves during the first week of life.

Confusion remains regarding BS type 5 as there are two different mutations that produce a phenotype designated Type 5. One “type” of BS type 5, i.e., a severe antenatal form of BS (BS type 5), arises as a result of a mutation in MAGED2, which affects the delivery of NKCC2 to the luminal membrane of loop of Henle cells, which spontaneously resolves during the first week of life. The other type of BS type 5, now more clearly designated as “autosomal dominant hypocalcemia with renal salt wasting” results from a gain-of-function mutation in the CaSR that blunts ROMK channel K efflux along with activity of the Na-K-2Cl cotransporter.

The simultaneous presence in GS of hypokalemia with hypomagnesemia and hypocalciuria in addition to the occurrence in early adulthood and a generally milder clinical presentation distinguishes GS from BS [1].

## 2. The GS and BS Phenotypic Overlapping

There is significant overlap in clinical manifestations between GS and BS and laboratory findings [2]. Mutations in the CLCNKB gene encoding the human voltage-gated chloride ClC-Kb (hClC-Kb) channel cause a classic type 3 BS, which shows phenotypic overlap with GS. Whereas mutations in CLCNKB are tied to clinical phenotype switching, the mechanism(s) is not well understood, and it is not always possible to correlate a genotype with severity of disease [3,4].

Lee et al. recently investigated a group of patients with chronic hypokalemic metabolic alkalosis [5]. They found wide variability in clinical phenotype, whereas variations in genotype did not correlate with either clinical course or electrolyte requirements. Although sex, genotypes, or the number of SLC12A3 mutant alleles did not predict severity or response to treatment, hypocalciuria and hypomagnesemia were useful markers to differentiate GS from classical type 3 BS [5]. Cheng et al., using a group of classic type 3 BS patients carrying homozygous missense mutations with well-described functional consequences and clinical presentations, found significant correlations of mutant chloride current activity with the age at diagnosis, plasma chloride concentration and urine calcium excretion rate [6]. These measurements were possible due to the rescue from accelerated degradation of expressed mutant protein by co-expression of barttin. Wojciechowski et al. noted that several cases of Dent disease 1 (DD1), another renal salt-wasting tubulopathy arising from mutations in the Cl^−^/H^+^ antiporter ClC-5, had atypical hypokalemic metabolic alkalosis and hyperaldosteronism, findings usually associated with BS [7]. This overlap of DD1 and BS phenotypes suggested that barttin might regulate ClC-5 transport and they found, using barttin cotransfection, that barttin impaired ClC-5’s complex glycosylation. Of note, pathologic barttin mutants are responsible of BS type 4a and the overlap of DD1 and BS phenotypes, despite clearly different genetic defects; this suggested that their overlapping phenotypes are a product of a disturbance of pathways common to both, in particular, intracellular glycosylation.

De Jong et al. investigated the effects of several different mutations found in GS patients to uncover the underlying pathogenic mechanism [8]. Specifically, they examined the effects of NCC mutations of differing types (missense, frameshift, nonsense, and splice-site mutations) at various portions of the entire protein coding sequence on metolazone-sensitive Na^+^ uptake, subcellular localization, and glycosylation of human NCC expressed in *Xenopus laevis* oocytes [8]. The GS mutations examined either had improperly glycosylated nonfunctional proteins that failed to exit the protein secretory pathway or functional mutants that were normally glycosylated but with partly impaired delivery to the plasma membrane. De Jong et al.’s results documented that mutations in NCC are associated with changes in NCC processing and that these defects are part of the underlying pathogenic mechanism in GS. However, although providing additional insight into the processing effects of the mutations, their report did not provide an explicit mechanism linking these NCC defects to the myriad of clinical effects that characterize GS.

Two other recent clinical studies investigating two unrelated ion transport mutations are relevant to our hypothesis, as they have been linked to changes in Golgi pH and glycosylation. Khayat et al. reported a multigenerational nonsyndromic intellectual disability that is the result of mutation in the alkali cation/proton exchanger gene SLC9A7 (also commonly referred to as NHE7) located on human X chromosome [9]. The gene is widely transcribed in various secretory tissues with prominent enrichment in the trans-Golgi network and post-Golgi vesicles. Mass spectrometry analysis showed an abnormal N-glycosylation profile for transferrin, a clinical diagnostic marker for congenital disorders of glycosylation. This then led Khayat et al. to conclude that misregulation of TGN/post-Golgi pH homeostasis and glycosylation of exported cargo likely underlay the cellular pathophysiology associated with nonsyndromic form of X-linked intellectual disability linked to a mutated SLC9A7.

Another study by Bastug et al. [10] was the case of a six-year-old girl who presented with hypokalemic metabolic alkalosis that had prompted an initial diagnosis of BS. However, the patient failed to thrive, and upon being reexamined two years later, slit-lamp cornea examination found punctate needle-shaped crystals and her leukocyte cystine content level was 15.1 mg/mL. The cystinosis diagnosis was confirmed by finding a homozygous c.853-1G>A novel splice mutation in cystinosin. Bastug’s review of the literature showed that cystinosis with hypochloremic metabolic alkalosis was first reported in a five-year-old boy by Berio in 1978 [11] with only 10 further cases with nephropathic cystinosis initially presenting as BS. Nephropathic cystinosis is a lysosomal disorder caused by functional defects of CTNS, a lysosomal 7-transmembrane protein H^+^-driven cystine transporter that mediates cystine efflux into the cytosol. Taranta et al. characterized cystinosin protein isoforms resulting in alternative splicing of exon 12, which directs the protein to other cell compartments, including the plasma membrane, the Golgi apparatus, the endoplasmic reticulum (ER), and cytosolic vesicles resembling endosomes [12].

## 3. RAS in Gitelman’s and Bartter’s Syndromes

Activation of RAS and production of Ang II lead to adverse cardiovascular remodeling as well as many other cardiovascular pathologies. RAS activation, as shown by the increased Ang II levels, typically accompanies increasing heart failure and systemic and tissue RAS dysregulation, which contribute strongly to end-stage heart failure via maladaptive cardiac remodeling, cardiac hypertrophy, apoptosis, fibrosis, and endothelial dysfunction [13].

Ang II-mediates up-regulation of Apelin (APLN), which in turn up-regulates ACE2, leading to conversion of Ang II by ACE2 into the protective Ang 1-7 peptide [14,15]. The APLN pathway has emerged as a major peptide hormone pathway with APLN widely expressed in mammals [16] and ACE2 is an important target of APLN action in the vasculature [17]. The regulatory loop also functions such that apelin 13 is required for ACE2 expression [18,19,20]. ACE2 clearly serves as a major negative RAS regulator in part by converting Ang II into the vasculoprotective, antiatherosclerotic, antioxidant, and anti-inflammatory peptide, Ang 1-7. 

Using GS/BS patients as nature’s experiments, we found them to be a human model of endogenous Ang II antagonism with activated RAS and high Ang II levels, yet blunted cardiovascular effects [1]. These patients, despite increased Ang II levels, have normotension or hypotension and lack of cardiovascular remodeling in terms of lack of cardiac left ventricular hypertrophy and carotid intima-media thickness, reduced intracellular calcium signaling, reduced Rho kinase activity, and activation of antiatherosclerotic, anti-inflammatory, and antioxidant defenses [1]. 

Of particular relevance is our demonstration in GS/BS patients of increased levels of both ACE2 and Ang 1-7, the levels of which were directly correlated, in contrast to either hypertensive or healthy subjects [21]. The ACE2/Ang 1-7 system was also shown be protective in hyperoxic lung injury [22]. Clearly, RAS has at least two counterregulatory axes, ACE/Ang II and ACE2/Ang 1-7, and their balance appears fundamental in maintaining cardiovascular homeostasis [23].

## 4. ACE2 and Chloroquine Effect

Of particular interest are the reports detailing the effects of chloroquine and other members of this class of drugs. Chloroquine (CQ), N4-(7-chloro-4-quinolinyl)-N1,N1-diethyl-pentane-1,4-diamine) is a weak base that, when unprotonated, can diffuse across membranes and accumulate in acidic cellular compartments. In these compartments, it is protonated and becomes trapped, causing elevated pH and swelling. For example, Basque et al. [24] examined multiple lysosomotropic drugs that alkalinized the trans-Golgi network (TGN)/endosome system and their effects on TGFβ production, which requires precursor protein cleavage in the TGN/endosomes for activity. They found that chloroquine, hydroxychloroquine, and azithromycin all suppressed processing of pro-TGFβ, the TGFβ precursor protein and reduced production of mature bioactive TGFβ. Chloroquine may also directly affect other cellular trafficking processes as well. Kavaliauskiene et al. reported chloroquine interferes with Shiga toxin subunit StxA1 translocation, potentially via effects on the Sec61 channel, as its knockdown also protects cells against Shiga toxin [25]. Sec 61 is part of the protein translocon, an ensemble of proteins involved in the ER targeting of precursor polypeptides [26]. McGill et al. reported that low dose chloroquine in patients with metabolic syndrome resulted in decreased blood pressure [27] as well as a decrease in JNK activation. Li et al. showed that MLN4760 an ACE2 inhibitor abolished captopril-induced blockade of JNK phosphorylation along with proinflammatory cytokines secretion and activation of p38MAPK and ERK1/2 [28]. These findings all suggest that chloroquine’s effect may be mediated via effects on ACE2.

## 5. Golgi-localized Glycosylation: The Importance of Endosomal pH 

Endosomes have an acidic interior due to the activity of a proton pump, and endosomal acidification is closely interlinked with endosomal, intracellular messenger (nicotinic acid adenine dinucleotide phosphate, NAADP)-mediated calcium release via a specific channel—the two-pore channel TPC2 [29]. Inhibition of the proton pump prevents calcium release from endosomes and reduction of extracellular calcium blocks endosomal acidification [29]. These processes have been shown to be critical for SARS-CoV-2 entry in the cell, which has been shown to be blocked when the proton pump is inhibited and, at least in part, explained by the reduced/lack of the intracellular messenger’s NAADP activity on TCP2 [29]. 

Kellokumpu suggested that Golgi-localized glycosylation is a pH-sensitive process [30]. For example, Axelsson et al. found that treatment with NH4CL caused an inhibition of O-glycan synthesis that paralleled the mislocalization of several glycosylation enzymes into endosomes with no effect on Golgi morphology [31]. Later, Kellokumpu et al. used chloroquine to change intracellular pH and found a 0.2 unit increase in pH interfered with both mucin type O-glycosylation and terminal a-2,3-sialylation of N-linked glycans without changing overall Golgi morphology [32]. Vincent et al. reported that in addition to chloroquine inducing elevations of endosomal pH, it interfered with terminal glycosylation of ACE2, the SARS-CoV cellular receptor, negatively influencing the virus receptor binding, resulting in the inhibition of infection [33].

## 6. The Basis for Gitelman’s and Bartter’s Syndrome Phenotypes

Looking at all the above-mentioned reports led us to think about some of the effects noted and we were specifically struck by the blood pressure effects and the noted changes in ACE2 glycosylation. We have sought to understand the basis for the disparate group of clinical effects that characterize GS and BS, as well as the differing levels of clinical severity that both GS and BS can exhibit. We have sought to understand how those clinical effects relate to the mutations in ion transport identified as causal in these syndromes. 

**Figure 2:**
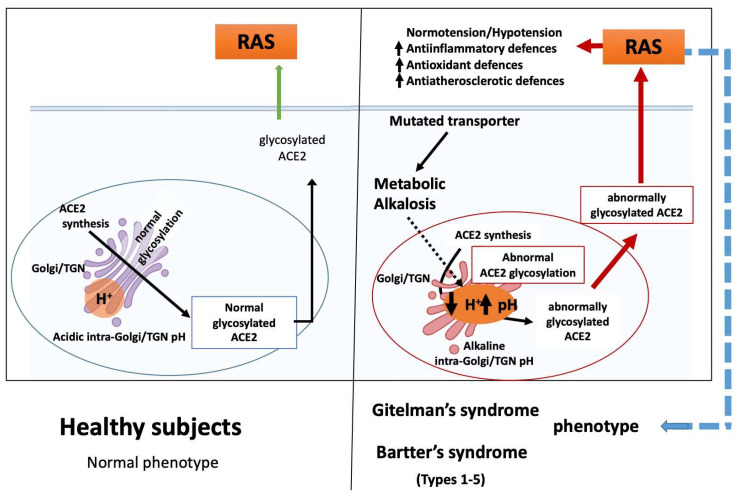
ACE2 protein glycosylation in a healthy subject and altered ACE2 protein glycosylation in GS/BS driven by changes in the pH of the TGN/endosome system with resulting effects on RAS.

We would like to suggest that the linkage between the specific gene defects identified in GS and BS and the myriad of alterations noted in clinical effects may be the result of aberrant glycosylation driven by altered TGN/endosome system acidification caused by the chronic metabolic alkalosis induced by their genetic defects. We think that either ion transport issues or chronic state of metabolic alkalosis in GS/BS patients, jointed with the blunted/reduced intracellular calcium signaling due to reduced second messenger induced intracellular calcium release [1], drive changes in the pH of the TGN/endosome system and result in altered ACE2 protein glycosylation (Figure 2).

The blood pressure effects, alongside the activation of antiatherosclerotic, anti-inflammatory, and antioxidant defenses found in GS and BS patients [1], are mirrored by chloroquine effects. This suggests that altered glycosylation of ACE2, found upon chloroquine treatment, occurs in GS/BS and drives the multiplicity of GS/BS effects. The central role of ACE2 in the counterregulatory system found in RAS then suggests that the effect of altered glycosylation is apt to be amplified by the RAS system. Similar clinical findings hint at common pathways for chloroquine and GS/BS effects with respect to cardiac electrophysiology. The recent surge of interest in hydroxychloroquine or chloroquine has led to the U.S. FDA issuing a safety announcement regarding their use being associated with QT interval changes and ventricular tachycardia [34]. These types of cardiac effects have also been found associated with GS/BS patients [21,35,36]. A mechanism whereby pH-driven altered glycosylation of ACE2 TGN/endosome system, combined with amplification by RAS, provides an explanation as to how GS and BS phenotypes can exhibit overlapping clinical findings despite their different mutations.

Of note, this mechanism also potentially provides an explanation for our findings regarding the absence of COVID-19 in GS/BS patients despite having an increased level of ACE2 [21], which has been shown to be the entry point for SARS-CoV-2. Increased levels of ACE2 have been linked to increased COVID morbidity and mortality [37] and thus should presumably result in an increased susceptibility to COVID-19 infection in GS/BS patients. However, our cohort of GS and BS patients provided evidence against that hypothesis, as a telephone survey of our over 100 GS and BS patients, all from Northern Italy (Veneto, Lombardia, and Emilia Romagna), the hotspots of the COVID-19 pandemic in Italy, found none of them infected with COVID-19 [38,39]. The very low incidence of these rare diseases limited our enrollment and the result of our survey was not significant (95% Confidence Interval (CI) 0–3%) using the reported COVID-19 prevalence in Northern Italy (0.65%, 95% CI 0.6–0.7%). However, given the likely COVID-19 underreporting [40], our result becomes significant (Pearson’s Chi-squared test *p* = 0.004) compared to the estimated true COVID-19 prevalence in Northern Italy (8.7%, 95% CI 8.7–8.8%) [41].

An altered terminal glycosylation of ACE2 in the TGN/endosome system caused by the gene mutation induced chronic metabolic alkalosis of these patients in addition to the altered intracellular calcium signaling due to a blunted second messenger induced intracellular calcium release, both of which increase endosomal pH [29], might reproduce the same pH-dependent effect on ACE2 glycosylation and resulting inhibition of Sars-CoV-2 by CQ and NH_4_Cl [8] and presumably COVID-19 infection. Cheng et al. just published an article that argues that we should focus on increasing ACE2 as a method of treating COVID-19 [42]. Similar studies on the use of human recombinant soluble ACE2 (hrsACE2) [43] support these findings. hrsACE2 has already passed through phase I and II clinical trials (NCT00886353, NCT01597635) for acute respiratory distress syndrome and has received regulatory approval (NCT04335136) for continued study in the fight against COVID-19 [44].

## 7. Conclusions

The proposed mechanism of ACE2 alterations in GS/BS requires much more intensive investigation and a study is ongoing in our laboratory to directly examine the terminal glycosylation of ACE2 in GS/BS patients to further strengthen our hypothesis. The linkage between the specific ion transport gene defects and the noted lack of correlation between genotype and phenotype in these patients might be the result of altered TGN/endosome system acidification causing aberrant glycosylation, which is then amplified by the RAS system. In other words, their pH enotype drives their phenotype.
